# Effect of Long-Term Aging on Fatigue and Thermal Cracking Performance of Polyphosphoric Acid and Styrene–Butadiene–Styrene-Modified Bio-Blend Bitumen

**DOI:** 10.3390/polym15132911

**Published:** 2023-06-30

**Authors:** Haitao Wang, Zhongming Du, Guiyong Liu, Xiaofeng Luo, Chunlu Yang

**Affiliations:** 1 China Construction First Group Corporation Limited, Beijing 100071, China; wanght@cscec.com (H.W.); cscecduzm@163.com (Z.D.); 2010391148@st.gxu.edu.cn (X.L.); YangchunluCCFGC@126.com (C.Y.); 2 School of Civil Engineering and Architecture, Guangxi University, Nanning 530004, China

**Keywords:** bio-blend bitumen, VECD model, fatigue properties, thermal cracking performance, microtopography

## Abstract

Polyphosphoric acid (PPA) and styrene–butadiene–styrene (SBS) were adopted to produce PPA-SBS-modified bio-blend bitumen, which achieved excellent mechanical performance. However, its long-range performance, such as the fatigue and thermal cracking behavior under long-term thermal oxidation, is not well understood. Therefore, a pressure aging vessel (PAV) system was applied to simulate the aging behavior of the bitumen under the action of thermal oxidation. Then, a linear amplitude sweep (LAS) test combined with a viscoelastic continuum damage (VECD) model was applied to investigate the fatigue properties of the bitumen. Moreover, a bending beam rheometer (BBR) test was conducted to evaluate the thermal cracking resistance of the bitumen before and after PAV aging. Meanwhile, an atomic force microscope (AFM) was applied to observe the microscopic topography. The results show that the original compound-modified bitumen can bear more fatigue damage than that of the control bitumen at the failure point, and it also has excellent fatigue resistance at 2.5%, 5%, 7.5%, and 10% applied strain. Moreover, the VECD model can accurately predict the fatigue life of the bitumen under different applied strains. The variation ratio of stiffness modulus for the compound-modified bitumen is below that of the control bitumen after PAV aging, so it shows a better anti-aging performance. Finally, the AFM test shows that PPA and bio-bitumen decrease the heterogeneity of the bitumen, reducing the difference between phases.

## 1. Introduction

Bitumen (which is called asphalt in the US) has become the main cementing material for road construction in terms of its excellent performance and easy maintenance [1,2]. Meanwhile, with the construction and maintenance of pavement, the demand for bitumen is still very huge [3]. However, bitumen is a non-regenerated resource. Therefore, with the consumption of crude oil, the lack of bitumen will have a huge adverse impact on road construction.

At the same time, biomass-regenerated materials have the advantages of environmental protection and sustainability [4], and the materials are possibly regenerated from grass [5], straw [6], manure [7], waste wood, and food scraps [8]. Therefore, it has gradually become the research hotspot of new pavement cementitious materials. Moreover, the usage of regenerated bio-materials in road construction can not only promote sustainability but also provide an effective way to dispose of biomass waste. Hence, bio-bitumen has a promising prospect under the background of advocating green and sustainable development. However, the inherent defects of the bio-bitumen are a direct obstacle to its usage in road engineering [9,10,11]. Therefore, how to improve its mechanical properties has become an urgent technical problem in the application of bio-bitumen. The previous research confirmed the three-dimensional elastic network of SBS and the reaction between PPA and bio-bitumen, improving the performance of the compound-modified bitumen and making the usage of the bio-bitumen possible [12,13,14]. However, there are few studies to investigate the long-range behaviors of compound-modified bitumen, although the long-term performance of bitumen mainly determines the lifetime and maintenance cost of the road, especially the fatigue resistance and low-temperature performance during the long-term service, so the fatigue and thermal cracking behavior under thermal oxidation are not well understood.

Fatigue and low-temperature cracking are the main causes of bitumen pavement failure. In the mixture system, using bitumen as the binder, its resistance to fatigue and low-temperature creep are particularly important [15]. In addition, the aging of bitumen has a significant effect on its anti-fatigue and thermal cracking properties [16,17,18]. In the early stage, SHRP proposed the use of fatigue factor ($G^{*}\cdot \sin\delta$) to characterize the anti-fatigue performance of bitumen. However, there are some differences in load conditions between the sweep test using a dynamic shear rheometer and the actual field. Therefore, the researchers believe that they cannot accurately characterize the anti-fatigue properties of bituminous materials [19]. To this end, Johnson et al. proposed the LAS test. Compared with the traditional $G^{*}\cdot \sin\delta$, the loading cycles to failure (*N_f_*) proposed by the LAS test have better accuracy and practicability [20,21,22]. Many studies have been conducted on bitumen binders using the LAS test. Liu et al. found that 2% polyphosphoric acid +2% waste engine oil and 1% poly-phosphoric acid +4% waste engine oil could increase the *N_f_* of bitumen by 1000% and 1150%. Meanwhile, the combination of PPA and waste engine oil could also improve the self-healing ability of bitumen [23]. Yan et al. used the LAS test to explore the fatigue resistance of seven kinds of modified bitumen and found that the thermos-plastic elastomer modified bitumen has better fatigue resistance [24]. Although the fatigue and thermal cracking resistance of the bitumen under long-term aging effects have a great influence on service performance and life-cycle cost, there are few studies to characterize these properties. In addition, it is important to explore the perennial performance of bitumen for booting its practical usage in road construction.

To address this concern, the LAS test and BBR test were used to investigate the fatigue resistance at medium temperature and crack resistance at a low temperature of the bitumen before and after long-term aging. At the same time, AFM was used to investigate the microtopography of the bitumen and bitumen after long-term aging. This study contributed to providing support for the application and reference for performance optimization of PPA/SBS compound-modified bio-blend bitumen and also provides a reference for the research and development of similar sustainable high-performance materials and long-term performance verification.

## 2. Raw Materials and Experimental Methods

### 2.1. Raw Materials

The petroleum bitumen (PAS), Sehll-90, from Shell Bitumen Company (Tian Jing, China), was obtained as base bitumen. The technical properties are as follows: penetration: 86.6 dmm (25 °C, ASTM D 5); ductility: 69.3 cm (10 °C, ASTM D 113); softening point: 46.4 °C (ASTM D 36); and rotational viscosity: 440 mP·s (ASTM D 4402). The bio-bitumen (BAS) is regenerated from straw by fermentation and hydrogenation, and its technical properties are as follows: relative density: 1.240 g/cm^3^ (25 °C, ASTM D 70); kinematic viscosity: 240 mm^2^/s (ASTM D 2170); pour point: 24 °C (ASTM D 97); and flash point: 265 °C (ASTM D 92). The PPA used in this research is industrial grade and have relative H_3_PO_4_ content of 115%. The copolymer used in this study is a linear styrene-butadiene-styrene with 30/70 block ratio.

### 2.2. Sample Preparation

First, the PAS and BAS were heated to 145 °C, and they were preblended to create two kinds of sufficiently homogeneous bio-blend bitumens, which are 90% PAS: 10% BAS (BBB1) and 85% PAS: 15% BAS (BBB2). Secondly, the PAS and BAS mixture (latterly called bio-blend bitumen) was heated to 175 °C. Then, the SBS modifier was poured into the bio-blend bitumen and mixed at 5000 rpm for 40 min using a high-speed shearing machine (a stabilizer was added to control group in the final 10 min). Thirdly, the PPA was poured slowly into the molten mixture and sheared for 30 min at the same conditions (the control bitumen was sheared without adding PPA). Finally, the well-homogenized compound-modified bitumen was developed at 175 ± 5 °C for 1 h in an oven.

**Figure 1:**
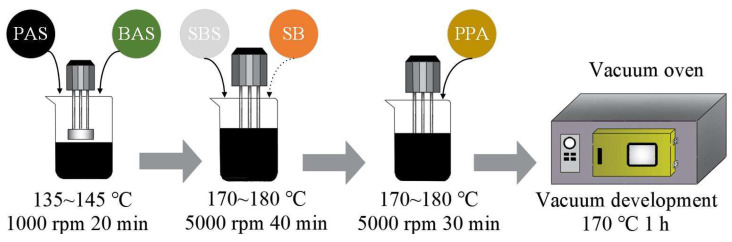
Preparation method of PPA-SBS-modified bio-blend bitumen.

**Figure 2:**
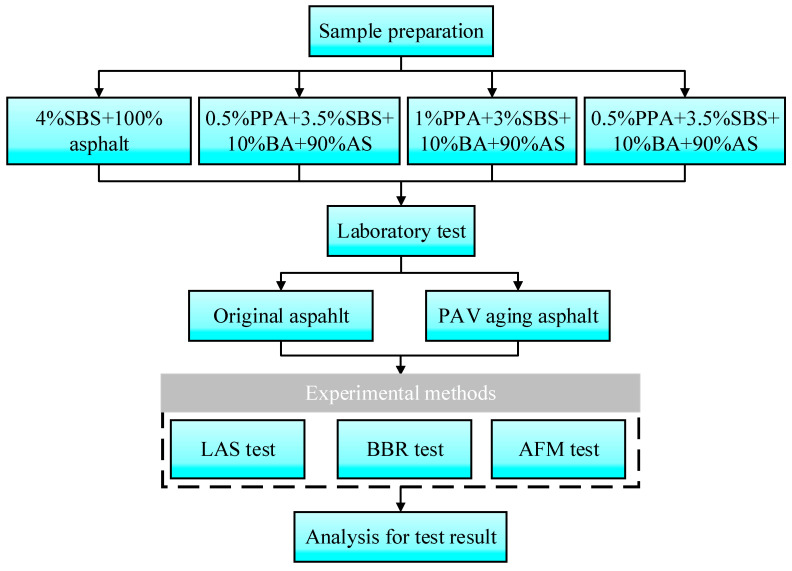
Flow diagram of the study.

The blending schemes of the compound-modified bitumen are as follows: the control bitumen consisted of 4% SBS and 100% PAS (A1). There are three blending schemes of compound-modified bio-blend bitumen, BBB1 plus 0.5% PPA and 3.5% SBS (A2), BBB1 plus 1% PPA and 3% SBS (A3), and BBB2 plus 1.5% PPA and 2.5% SBS (A4), which are obtained from our previous research [13,14]. Figure 1 and Figure 2 are diagrams of the preparation methods and flowchart of the research, respectively. 

### 2.3. Experimental Methods

#### 2.3.1. Long-Term Aging Process

A PAV system (Model 9300 from Prentex, Sunnyville, TX, USA) was used to characterize the qualitative changes of bitumen during long-term service. The test was conducted according to ASTM D 6521. Firstly, the bitumens were treated by a thin film oven at 163 °C for 5 h. Next, the residues were aged for 20 h in the PAV system. The temperature was 100 °C, and the pressure was 2.1 MPa.

#### 2.3.2. Dynamic Shear Frequency Sweep Test

Frequency sweep test was first applied to each bitumen sample using a dynamic shear rheometer (AR1500ex from TA Instruments, New Castle, DE, USA) with an 8 mm parallel plate and 2 mm gap. The test was conducted according to AASHTO TP 101-14 at 25 °C. The shear strain was controlled at 0.1%, and the frequencies were 0.2, 0.4, 0.6, 0.8, 1, 2, 4, 6, 8, 10, 20, and 30 Hz.

#### 2.3.3. Amplitude Strain Sweep Test

Amplitude strain sweep test is a method used to simulate the fatigue behavior of bitumen at medium temperature. The test temperature, equipment, and standard are the same as the frequency sweep test, but the shear strain is first increased from 0.1% to 1% and then linearly increased from 1% to 30% at a frequency of 10 Hz.

#### 2.3.4. Fatigue Properties Based on the VECD Model

The fatigue properties were calculated based on VECD theory according to the following steps [25].

1. Performance parameter α was used to evaluate the stress sensitivity of the undamaged sample. Firstly, the energy storage modulus was calculated according to Equation (1) through the phase angle and complex modulus recorded during the frequency sweep test. Then, the slope m-value and intercept b-value were obtained by fitting Equation (2). Finally, *α* was calculated by Equation (3).
  (1) $$G^{\prime }\left(\omega \right)=G^{*}\left(\omega \right)\cos\delta \left(\omega \right)$$
  (2) $$\mathrm{lg}G^{\prime }\left(\omega \right)=\mathrm{mlg}\omega +b$$
  (3) $$\alpha =\frac{1}{m}$$
2. The damage intensity *D(t)* was used to evaluate the accumulated damage of the bitumens during the oscillating stress. And it was calculated according to Equation (4).
  (4) $$D(t)≅{\sum_{i=1}^{N}\left[\pi \gamma_{0}^{2}\left(C_{i-1}-C_{i}\right)\right]}^{\frac{\alpha }{1+\alpha }}{\left(t_{i}-t_{i-1}\right)}^{\frac{1}{1+\alpha }}$$
  (5) $$C(t)=\frac{G^{*}(t)}{G_{0}^{*}}$$
  where C(t) is the integrity index of the specimen over time; $\gamma_{0}$ is the strain applied; *t* is the time over the test; and $G_{0}^{*}$ is the initial complex modulus of the material.

$C_{1}$, $C_{2}$, were fitted according to Equations (6) and (7).

(6) $$C\left(t\right)=C_{0}-C_{1}D{\left(t\right)}^{C_{2}}$$

(7) $$\mathrm{lg}(C_{0}-C(t))=\mathrm{lg}C_{1}+C_{2}\mathrm{lg}D(t)$$

where $C_{0}$ is the initial integrity index of the material.

3. Damage accumulation (*D_f_*) was used to evaluate bearing capacity of fatigue damage of the bitumens.

In the early stage, Johnson et al. [25,26] and AASHTO TP 101-12 suggested that the cumulative damage when the integrity index of the specimen is reduced by 35% is taken as the failure point of the specimen. With the deepening of the research on bitumen fatigue behavior, Bahia and AASHTO TP 101-14 suggested that the cumulative damage corresponding to the peak shear stress should be taken as the failure point [27]. Therefore, this method is also adopted in this study, and the failure point (*D_f_*) of the specimens was calculated according to Equation (8).

(8) $$D_{f}={\left(\frac{C_{0}-C_{peak}}{C_{1}}\right)}^{\frac{1}{C_{2}}}$$

where $C_{peak}$ is the integrity index corresponding to the peak shear stress of the material.

4. Finally, the loading cycles to failure (*N_f_*) of each specimen were calculated according to Equation (9).
  (9) $$N_{f}=A\gamma^{-B}$$
  (10) $$A=\frac{f{\left(D_{f}\right)}^{1+(1-C_{2})\alpha }}{(1+(1-C_{2})\alpha ){\left(\pi C_{1}C_{2}\right)}^{\alpha }}$$
  (11) B=2α
  where f is the frequency, 10 Hz.

The specific operation of LAS test was carried out according to AASHTO TP 101-14 at 25 °C using a parallel plate with diameters of 8 mm and gap of 2 mm [25]. 

#### 2.3.5. Bending Beam Rheometer (BBR)

The low-temperature properties of original bitumens and bitumens after PAV aging were investigated using BBR test according to ASTM D 6648. The creep deformation of the specimen under a constant load of 980 ± 50 mN was recorded at −12 °C, −18 °C, and −24 °C, respectively. Then, the stiffness modulus and its variation rate (m-value) of the specimen at 60 s were adopted as the thermal cracking properties to characterize low-temperature creep of the compound-modified bitumens.

#### 2.3.6. Atomic Force Microscopic (AFM)

Many studies have indicated that the microscopic surface flatness is related to the intermolecular homogeneity of bitumen [28,29,30]. Therefore, an AFM (FastScan from BRUKER, Billerica, MA, USA,) was applied to observe the microtopography of the compound-modified bitumens. The molten bitumen was poured into a metal container with an inner diameter of 16 mm and a height of 3.5 mm; Then, the specimen was swept by tapping mode within an area of 15 μm × 15 μm. Finally, topography process was carried out with NanoScope version 1.4 analysis software.

## 3. Results and Discussion

### 3.1. Analysis of Frequency Sweep Test

**Figure 3:**
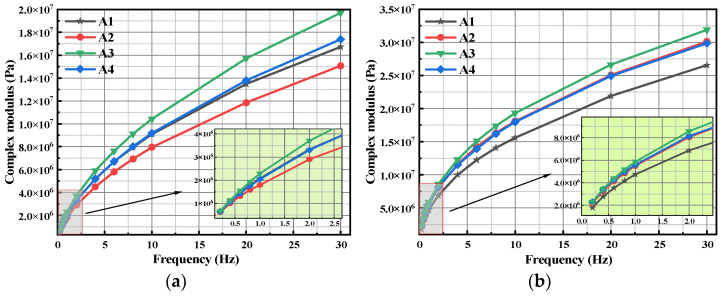
Complex modulus for different bitumens: (**a**) original bitumen; (**b**) PAV-aged bitumen.

Figure 3 shows the frequency sweep test results of each bitumen. As can be seen from Figure 3a, at a lower shear frequency, A2 has the lowest complex modulus, while A4 has no significant difference with A1, and A3 is the largest but does not show a significant difference on the whole. With the increase of frequency, the complex modulus of the bitumens gradually increases, and the difference of the complex modulus between the four bitumens also increases obviously, but the variation trend is the same as that at the lower frequency. Our previous studies have shown that PPA and bio-bitumen can be esterified so that they can provide certain mechanical properties [12,13,14]. Therefore, A2 does not have enough esterification products to compensate for the loss of mechanical strength while reducing SBS content, so its complex modulus is lower than A1. Although the SBS content of A4 is the lowest, the comprehensive content of PPA and bio-bitumen is the highest, so the complex modulus of A4 is not obviously below that of A1. In addition, A3 has the highest complex modulus, which can be attributed to it having an optimal mix scheme of SBS, bio-bitumen, and PPA, so it has the largest complex modulus at any frequency.

The complex moduli for each bitumen after PAV aging are shown in Figure 3b. After PAV aging, the complex moduli of each bitumen increase significantly. In addition, the increasing rates for the compound-modified bitumen are larger than that of the control bitumen. The reason can be attributed to the fact that bio-bitumen contains a larger amount of light components, and it is volatilized and condensed under the action of high temperature and oxygen, so their complex moduli are significantly increased. The variation trend and difference of complex modulus of the three compound-modified bitumens are roughly the same as that of their original bitumens.

### 3.2. Analysis of Stress–Strain Relationship in Amplitude Strain Sweep Test

**Figure 4:**
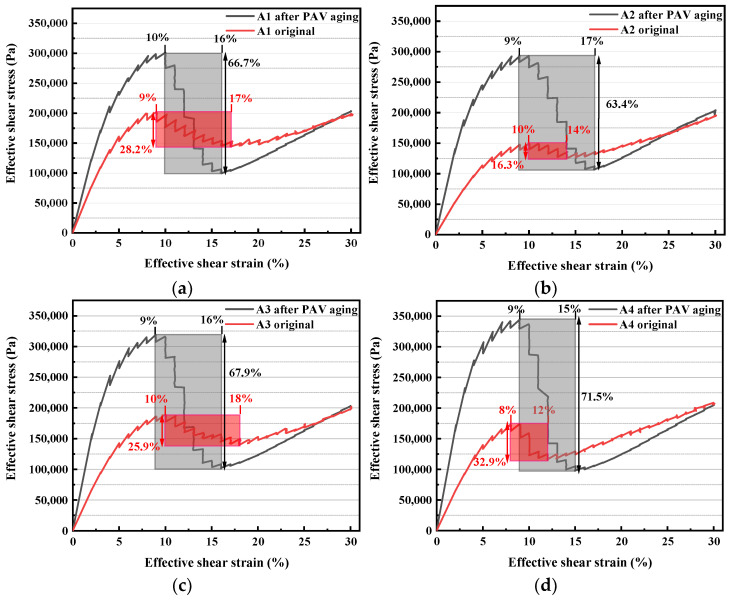
Strain–stress curve for different bitumen: (**a**) A1; (**b**) A2; (**c**) A3; (**d**) A4.

Figure 4 shows the stress–strain curves of LAS tests of each bitumen. As for the original bitumen, the maximum shear stress of the three compound-modified bitumens was lower than that of the control bitumen. Among them, A2 has the largest reduction rate, which is 25% lower than that of A1, followed by A4 and A3, which are 13% and 6.25% lower than that of A1, respectively. The reason for the lower reduction rate of A3 and A4 may be that the esterification products of PPA and bio-bitumen provide certain mechanical strength. While A3 has a better proportion of PPA, BA, and SBS, the esterification products and SBS synergistically provide better mechanical strength, resulting in A3 having less reduction rate of the maximum shear stress than A1. 

It is worth noting that the bitumens can still recover the positive stress–strain relationship after obvious fatigue failure, and with the increase of strain, the shear stress gradually exceeds or equals the maximum shear stress before fatigue failure. The shadow part in the stress–strain curve is the marked fatigue failure zone, which is defined as the region consisting of the maximum shear stress point before the fatigue failure and the minimum shear stress point. Therefore, it is speculated that the fatigue failure of the SBS and compound-modified bitumen in this study may be composed of three aspects: (1) stress fatigue of SBS elastomer under oscillatory shear stress, (2) fatigue failure of bitumen, and (3) fatigue failure of esterification products of PPA and bio-bitumen. Specifically, at the initial stage of fatigue failure, SBS, bitumen, and esterification products jointly provide mechanical strength. However, as the fatigue failure of the inelastic phase of bitumen tends to be complete, the proportion of mechanical strength provided by the inelastic phase decreases significantly. At this time, SBS are dominant, so the stress–strain curve of the specimen recovers a positive correlation, but the slope is below that of the initial stage.

Moreover, the esterification products of PPA and bio-bitumen have a certain alleviating effect on the fatigue failure of compound-modified bitumen. The minimum shear stress of the SBS control group is reduced by 28.2% compared with the peak shear stress. In contrast, A2 and A3 are 16.3% and 25.9%, respectively, while A4 may have a large reduction of 32.9% due to the lower content of SBS. The fatigue failure of the four bitumens starts at a strain point of 9% but stops at different strain points. The fatigue failure interval of A1 is from 9% to 17%, A2 is from 10% to 14%, A3 is from 10% to 18%, and A4 is from 8% to 12%. The reasons may be as follows: PPA is grafted onto bio-bitumen through an esterification reaction, thus improving the crosslinking effect between molecules in bitumen. At the same time, the collaborative spatial network of SBS makes the fatigue failure of A2 smaller and tends to be complete earlier, so its failure strain interval is smaller. However, the alleviating effect of the elastic spatial network on fatigue failure decreases along with the reduction of SBS concentration, so the fatigue failure of A3 increases, and the failure strain interval increases. With the ulteriorly decrease of SBS concentration, the alleviating effect of the elastic spatial network on fatigue failure is significantly reduced, resulting in the increase of the fatigue failure of the inelastic phase of A4 and tends to be complete in the lower strain range.

For the PAV-aged bitumen, the peak shear stress and stress reduction rate (i.e., the height of the fatigue failure zone in the stress–strain curve) of the four bitumens are significantly higher than those of the original bitumen. In addition, the fatigue failure zone of the four bitumens after aging with PAV is different. Firstly, the difference between the stress reduction rate of the compound-modified bitumen and the stress reduction rate of A1 is not as significant as the original bitumen, but the overall variation trend is similar to the original bitumen. Secondly, the strain interval of fatigue failure of the compound-modified bitumen shows an increasing trend after PAV aging.

### 3.3. Analysis of Fatigue Properties in VECD Theory

#### 3.3.1. Analysis of VECD Curve

**Figure 5:**
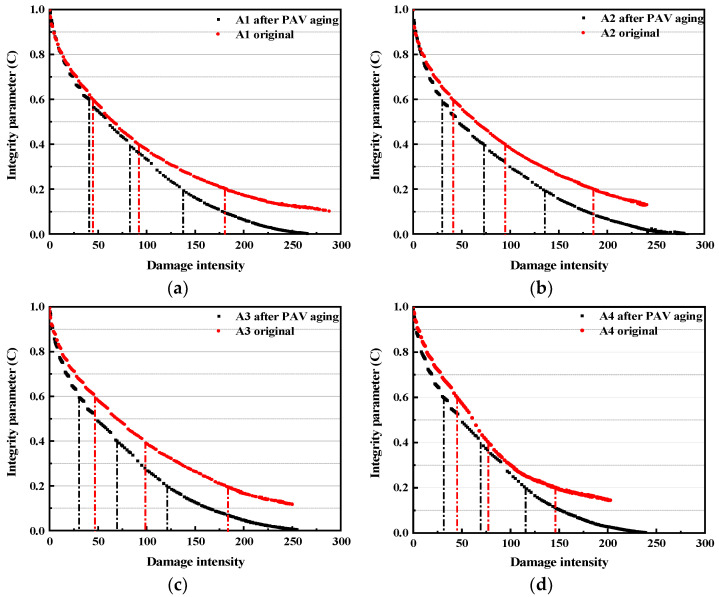
VECD curve for different bitumen: (**a**) A1; (**b**) A2; (**c**) A3; (**d**) A4.

Figure 5 shows the VECD curves of each bitumen. When *C* = 1, it indicates that the specimen has not been damaged; when *C* = 0, it indicates that the specimen has been completely damaged [25,31,32]. As for the original bitumen, the cumulative damage intensity (*D*) of A2 and A3 under the same integrity parameter (*C*) is not significantly different from that of A1, while the *D*-value of A4 after *C* = 0.6 is significantly lower than that of A1. It is worth noting that the *C*-value of the four bitumens under the maximum *D*-value condition is still not zero, indicating that the specimen is not completely failing. Moreover, the *C*-value of the compound-modified bitumen under the maximum *D*-value is slightly greater than A1 (corresponding to the esterification products that have a certain protective effect on the fatigue damage of SBS). Moreover, the maximum *D*-value of the compound-modified bitumen is slightly smaller than that of A1. This might be attributed to the mechanical strength afforded by the esterification products of PPA and BAS. Among them, the maximum *D*-value of A2 is 232, A3 is 253, and A4 is 208. Combined with the stress–strain curves of the bitumens, it can be inferred that the maximum cumulative damage may be related to the strain interval of the fatigue failure zone. Specifically, A1 and A3 have larger fatigue failure strain intervals, so their maximum *D*-values are larger, while A2 and A4 have smaller fatigue failure strain intervals, so their maximum *D*-values are smaller. Meanwhile, although A4 and A2 have the same interval of failure strain, the maximum *D*-value of A4 is still smaller than that of A2, which may be attributed to the smaller failure strain of A4. 

After PAV aging, the *C*-value of the four bitumens is significantly reduced, and its lowest value is close to zero, indicating that fatigue failure has occurred completely. It is different from A1 that the maximum *D*-value of the compound-modified bitumens is larger than that of their unaged bitumen, indicating the compound-modified bitumen bore more fatigue damage. However, some researchers believe that it is still difficult to evaluate the fatigue performance of different bitumen materials simply through the VECD curve [31,32]. Therefore, the damage accumulation (*D_f_*) is introduced to further discuss the anti-fatigue property of the compound-modified bitumen binder.

#### 3.3.2. Analysis of Damage Accumulation

**Figure 6:**
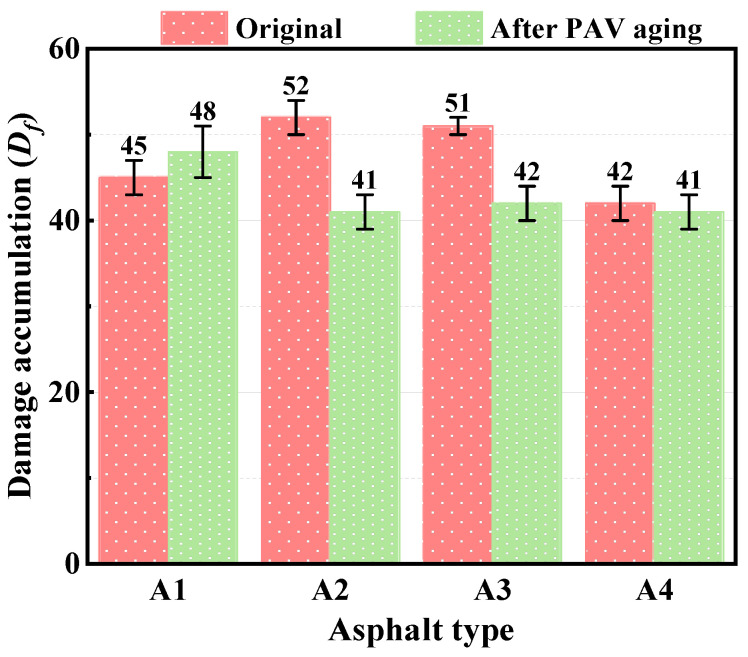
*D_f_* for different bitumen before and after PAV aging.

The damage accumulation at fatigue failure (*D_f_*) of each bitumen is shown in Figure 6. For unaged samples, *D_f_*-values of A2 and A3 are higher than that of A1, while A4 is slightly lower than A1, which corresponds to the variation of the stress reduction rate of the bitumen in the stress–strain curve. The larger *D_f_*-value means that the bitumen specimens can withstand more damage before fatigue failure. Therefore, the esterification products of PPA and bio-bitumen can improve the resistance to fatigue damage to a certain extent. However, the *D_f_*-value of A4 is lower than that of A1, and this may be because of the lower SBS content.

After PAV aging, the *D_f_*-value of A1 is higher than that of its original bitumen, while the *D_f_*-value of the compound-modified bitumen is below that of the original bitumens, and A2 and A3 decrease the most. Long-term aging leads to the agglomeration of bitumen molecules, which results in the hardening of bitumen and significantly increased modulus [33,34]. However, there are many light components in the bio-bitumen used in this study, so the volatilization and agglomeration of light components after long-term aging led to the reduction of fatigue damage tolerance of the compound-modified bitumens. However, due to the high content of PPA and bio-bitumen, the esterification products have a certain alleviating effect on the *D_f_*-value reduction of A4. Therefore, the *D_f_*-value of A4 after PAV aging is only slightly smaller than that of its original bitumen.

#### 3.3.3. Analysis of the Strain Sensitivity Parameter

**Figure 7:**
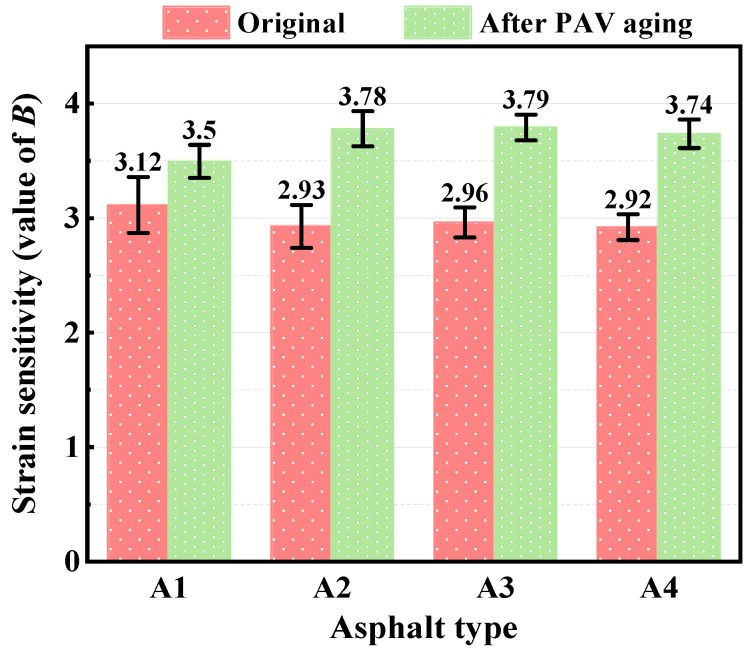
Strain sensitivity coefficient for different bitumen before and after PAV aging.

The strain sensitivity parameter (*B*-value) of each bitumen is shown in Figure 7. It can be seen from Equation (9) that *B*-value represents the sensitivity of loading cycles to failure (*N_f_*) to strain. The larger *B*-value is, the more sensitive the material is, which may result in a lower *N_f_*. Before aging, the *B*-value of the compound-modified bitumen is slightly lower than that of the A1, indicating that the esterification of PPA and bio-bitumen reduce the strain sensitivity of the bitumen. After PAV aging, the bitumens hardened and became brittle, resulting in the *B*-value of the four bitumens increasing significantly. Moreover, the increment in the *B*-value of the compound-modified bitumens is larger than that of A1, indicating that the fatigue life of the compound-modified bitumens is more sensitive to applied strain. 

#### 3.3.4. Analysis of Fatigue Life

**Figure 8:**
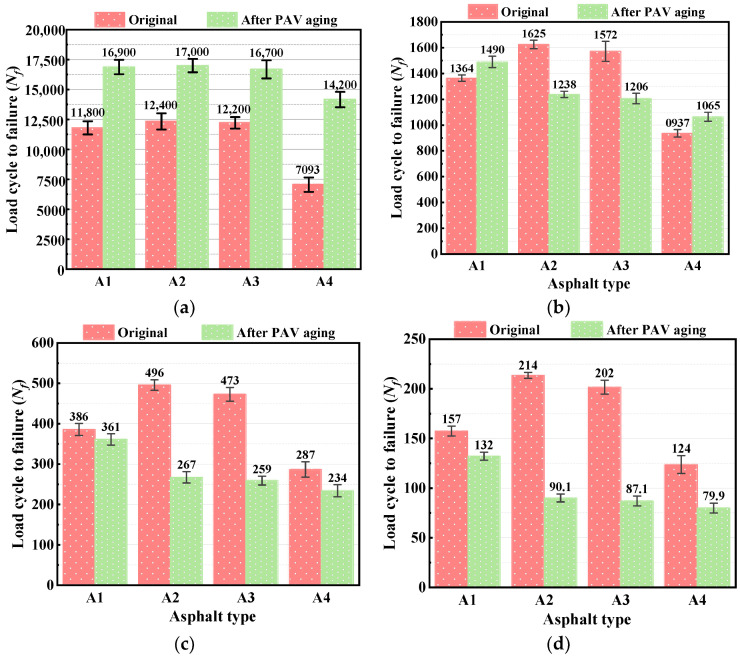
*N_f_* for different bitumen under various applied strains: (**a**) 2.5%; (**b**) 5%; (**c**) 7.5%; (**d**) 10%.

Figure 8 shows the loading cycles to failure (*N_f_*) of the original bitumen and bitumen processed by PAV aging under different applied strains. As can be seen from the figure, with the gradual increase of applied strain, the *N_f_* of each bitumen decreases significantly, indicating that the increase of strain level significantly aggravates the fatigue damage inside the bitumen. As for the original bitumen, the fatigue life of A2 and A3 is higher than that of A1 at any applied strain level, which also indicates that the esterification produced by the reaction of PPA and bio-bitumen plays a positive role in improving the fatigue life of bitumen. The fatigue life of A4 is significantly reduced; this can also be attributed to the low content of SBS.

At a lower applied strain (2.5%), the *N_f_* of the bitumens after PAV aging is significantly higher than that of their original bitumen. The reason may be that bitumen condenses after PAV aging so as to intensify intermolecular forces, resulting in less fatigue damage of the bitumens under the condition of a lower applied strain level. However, with the increase of applied strain, the *N_f_*-value of the PAV-aged bitumens decreases significantly. Meanwhile, the reduction of the *N_f_*-value of the compound-modified bitumens after PAV aging is obviously larger than that of A1, indicating the compound-modified bitumens have poor fatigue resistance after long-term aging under the condition of larger applied strain, which is consistent with the results of *B*-value. However, long-term aging has little effect on A4 with higher PPA and bio-bitumen content, and the *N_f_* of A4 is significantly lower than that of its original bitumen, only at 10% applied strain. The reasons may be as follows: (1) the SBS content of A4 is lower, and PAV aging further reduces its immediate elastic recovery, as shown in our previous research; (2) although the esterification produced by the reaction of PPA and bio-bitumen provides a part of the mechanical strength by improving the crosslinking effect between molecules, it is mainly composed of simple single bonds and intermolecular forces, so the fatigue-damage resistance is poor under larger applied strain.

**Figure 9:**
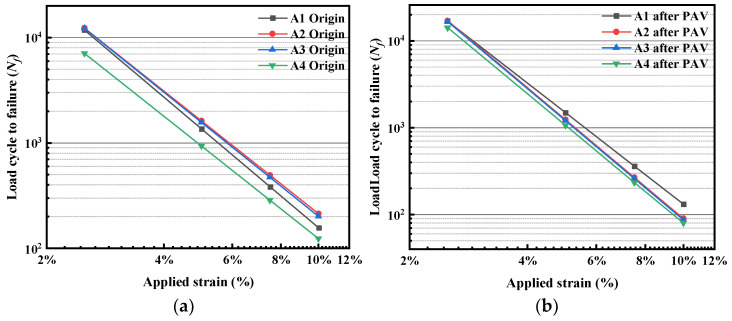
The fitting of log *N_f_*−og ε: (**a**) original bitumen; (**b**) PAV-aged bitumen.

In order to further explore the relationship between *N_f_* and the applied strain of each bitumen and to accurately predict the fatigue life of the compound-modified bitumen, the linear fitting of applied strain and *N_f_* was carried out using double logarithmic coordinates, as shown in Figure 9. The fitting results and predicting equations are listed in Table 1.

The slope of the predicting equation can represent the strain sensitivity. A larger slope means the sample is more sensitive to the increase of applied strain and the lower the *N_f_*. By comparing the slope of the predicting equation of the unaged samples, it is revealed that the slope of the compound-modified bitumens is below that of A1, and its variation trend corresponds with the *B*-value, which also indicates that the predicting equation can accurately characterize the fatigue properties of the bitumens. After PAV aging, the slope of each predicting equation is significantly improved, and the improvement of the compound-modified bitumen is larger, which is consistent with the previous analysis. 

In summary, the esterification products of PPA and BAS have a positive effect on the improvement of fatigue resistance of the bitumens under the condition of small applied strain, but its fatigue resistance is poor under the condition of larger applied strain. Therefore, the application of compound-modified bitumen is more inclined to the road and has high design speed and low generated strain.

### 3.4. Analysis of the BBR Test

The S- and m-value of each bitumen at different temperatures are shown in Table 2. As for original bitumen, the S-value of each group increased significantly with the decrease in temperature, while the m-value decreased gradually. The reason may be that the high content of light components that has higher glass transition temperature in bio-bitumen leads to the decrease of the low-temperature creep capacity of the compound-modified bitumens [35,36]. However, the S-value of the compound-modified bitumens did not further increase with the decrease in SBS concentration. Specifically, the SBS concentration of A3 was reduced by 25% compared with A2, but the S-value of A3 was not obviously increased compared with that of A2, and this variation also exists between A4 and A3. Therefore, it can be speculated that the esterification crosslinking of PPA and bio-bitumen leads to the form of the crosslinking network structure between molecules [37], which improves delay elasticity of the compound-modified bitumens, and reduces the negative effect of glass transition of light components in PAS and BAS on the thermal cracking resistance of the compound-modified bitumens.

**Figure 10:**
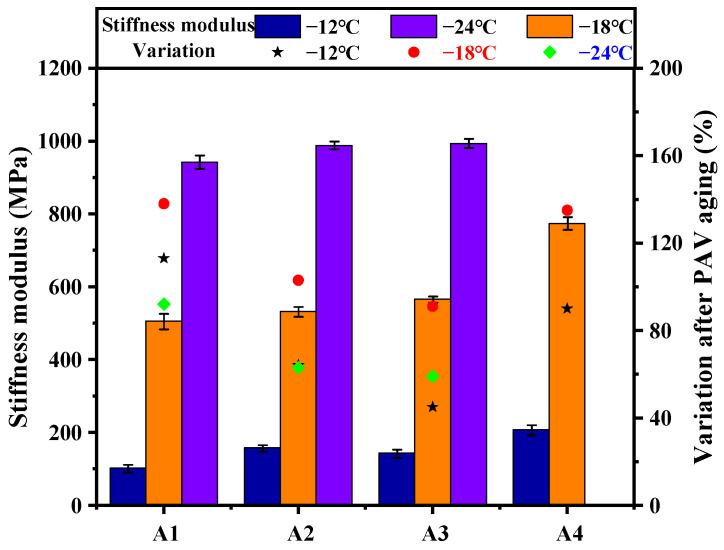
Stiffness modulus of the bitumens after PAV aging.

Figure 10 shows the stiffness modulus and variation rate of the bitumens after PAV aging. PAV aging significantly increased the S-value and decreased the m-value of all the bitumens. Among them, the S-value of A4 exceeded 1000 MPa at −24 °C. It does not meet the requirement of ASTM D 6648 for flexural creep stiffness values of measured materials need to be in the range of 20 MPa to 1 GPa. In addition, the S-value of the compound-modified bitumen was significantly larger than that of A1 at all temperatures, but the variation rate of the S-value for A2, A3, and A4 after PAV aging was below that of A1, which indicates that the esterification crosslinking of PPA and bio-bitumen improves the long-term aging resistance of the compound-modified bitumen at low-temperature.

### 3.5. Analysis of AFM Topography

The topography of the original bitumen has been discussed in detail and at great length in our previous research. Therefore, this paper presents only the main conclusion that the addition of bio-bitumen decreases the intermolecular heterogeneity of the compound-modified bitumen. Moreover, cracks tend to occur at the interface of phase to phase, so the greater the phase splitting, the worse the cracking resistance of the material [38]. Hence, the *N_f_* -value of the unaged compound-modified bitumen in the LAS test is higher than that of the control bitumen.

**Figure 11:**
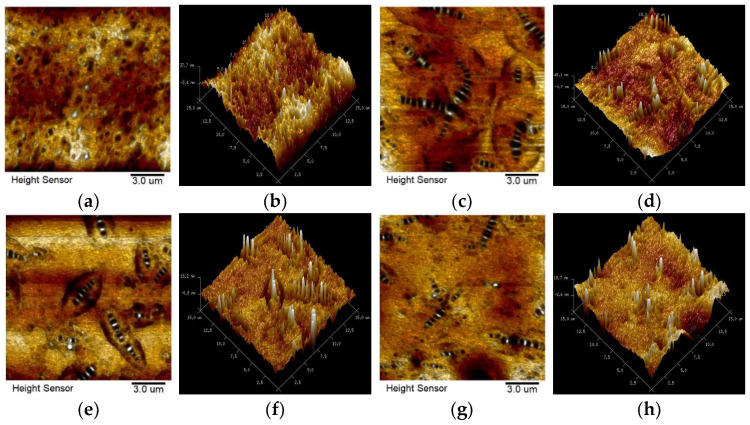
2D- and 3D-topography of the bitumens after PAV aging: (**a**,**b**) A1; (**c**,**d**) A2; (**e**,**f**) A3; (**g**,**h**) A4.

By comparing the surface topography of A2 before and after PAV aging in Figure 11, it is found that obvious contour embraces the periphery of the bee-like structure after aging, which indicates that aging improves the difference of the properties for the interface between the bee-like structure phase and the continuous phase. Hence, the fatigue and thermal cracking resistance of A2 decreased after PAV aging, which corresponds to the test results of LAS. However, the phase separation was reduced in A4 due to the increase of bio-bitumen and PPA concentration, and this contour also decreased significantly in A3, indicating that the addition of bio-bitumen and PPA reduces the intermolecular heterogeneity of the compound-modified bitumen. Similarly, in the LAS test, A3 and A4 have a lower reduction rate of *N_f_* -value than that of A2 after PAV aging.

## 4. Conclusions

To characterize the fatigue and thermal cracking properties of the environmentally friendly and sustainable bitumen material before and after the long-term thermal-oxidative effect, a linear amplitude sweep test was carried out based on the VECD model. In this way, the fatigue properties of PPA-SBS-modified bio-blend bitumen under various applied strains were studied. Moreover, the BBR test was adopted to investigate the thermal-creep properties of the bitumen and bitumen after PAV aging. The conclusions have been drawn are as follows:

1. Under the action of esterification crosslinking of PPA and bio-bitumen, the compound-modified bitumen can bear more damage. In addition, the fatigue damages of A2 and A3 are greater than that of the control bitumen at the failure point, but after long-term aging, the damage-bearing capacity of the compound-modified bitumen is significantly reduced;
2. The *N_f_*-value of unaged A2 and A3 at 2.5%, 5%, 7.5%, and 10% strains are higher than that of the control bitumen, which shows that the esterification produced by the reaction of PPA and bio-bitumen can effectively improve the fatigue life of the unaged bitumen. However, after long-term aging, the fatigue life of bitumens is only improved under the condition of small applied strain (2.5%);
3. The low-temperature performance of the compound-modified bitumen does not deteriorate further with the decrease in SBS content. Therefore, the esterification and crosslinking of PPA and bio-bitumen have positive effects on the low-temperature properties. The variation rate of the S-value for the compound-modified bitumen after PAV aging is significantly below that of the control bitumen, which means the compound-modified bitumen has better anti-aging performance in the BBR test;
4. After long-term aging, the surface morphology of the control bitumen is rough, which shows that the intermolecular heterogeneity increases significantly. In contrast, the surface topography of the compound-modified bitumen is relatively flat, but the number of its bee-like structure is increased. In addition, combined with the results of the medium linear amplitude test, it can be posited that PPA and bio-bitumen can delay the influence of aging on the fatigue life of compound-modified bitumen;
5. The esterified crosslinking provided by PPA and bio-bitumen has a benefit on the mechanical properties of bitumen, and it has been justified. However, the modification mechanism that we currently understand is superficial. Therefore, subsequent research should focus on the underlying mechanism and model establishment of the compound-modified bitumen.

## Figures and Tables

**Table 1:** The predicting equation of *N_f_* and ε of each modified bitumen.

Bitumen Type		Applied Strain (%)	*N_f_* (Times)	Predicting Equation
A1	Original	2.5	11,832	$\mathrm{lg}N_{f}=-3.116\mathrm{lg}\varepsilon -0.919$
		5	1364	
		7.5	386	
		10	157	
	After PAV	2.5	16,900	$\mathrm{lg}N_{f}=-3.500\mathrm{lg}\varepsilon -1.379$
		5	1490	
		7.5	361	
		10	132	
A2	Original	2.5	12,366	$\mathrm{lg}N_{f}=-2.928\mathrm{lg}\varepsilon -0.598$
		5	1625	
		7.5	496	
		10	214	
	After PAV	2.5	17,018	$\mathrm{lg}N_{f}=-3.781\mathrm{lg}\varepsilon -1.826$
		5	1238	
		7.5	267	
		10	90	
A3	Original	2.5	12,249	$\mathrm{lg}N_{f}=-2.962\mathrm{lg}\varepsilon -0.657$
		5	1572	
		7.5	473	
		10	202	
	After PAV	2.5	16,706	$\mathrm{lg}N_{f}=-3.792\mathrm{lg}\varepsilon -1.852$
		5	1206	
		7.5	259	
		10	87	
A4	Original	2.5	7093	$\mathrm{lg}N_{f}=-2.961\mathrm{lg}\varepsilon -0.829$
		5	937	
		7.5	287	
		10	124	
	After PAV	2.5	14,190	$\mathrm{lg}N_{f}=-3.737\mathrm{lg}\varepsilon -1.834$
		5	1065	
		7.5	234	
		10	80	

**Table 2:** The results of the BBR test.

Bitumen Type		−12 °C			−18 °C			−24 °C		
		S/MPa	Variation/%	m	S/MPa	Variation/%	m	S/MPa	Variation/%	m
A1	Original	48.411	↑ 113	0.448	212.224	↑ 138	0.331	492.217	↑ 92	0.240
	PAV	102.341		0.351	505.445		0.243	942.766		0.118
A2	Original	96.512	↑ 64	0.436	262.598	↑ 103	0.315	605.456	↑ 63	0.237
	PAV	158.152		0.314	532.166		0.221	988.145		0.104
A3	Original	98.902	↑ 45	0.442	296.726	↑ 91	0.323	623.775	↑ 59	0.232
	PAV	143.226		0.321	565.731		0.218	993.771		0.101
A4	Original	109.287	↑ 90	0.433	329.757	↑ 135	0.324	652.306	-	0.229
	PAV	207.851		0.291	774.167		0.197	1000+		-

Note: ↑ indicates an upward trend; + indicates that the data is greater than 1000.

## Data Availability

The data presented in this study are available on request from the corresponding author.
